# On Some Points in the Clinical History of Asthma

**Published:** 1859-11

**Authors:** Hyde Salter

**Affiliations:** Assistant Physician to Charing Cross Hospital


### ECLECTIC DEPARTMENT,

### AND SPIRIT OF THE MEDICAL PERIODICAL PRESS.

---

*On some Points in the Clinical History of Asthma.* By HYDE SALTER, M. D., F. R. S., Assistant Physician to Charing Cross Hospital.

### PHENOMENA OF A PAROXYSM.

Premonitory and initiatory symptoms.—Drowsiness, dyspeptic symptoms, headache, excitability, profuse diuresis, neuralgic pains.—Time of attack, the early morning; why?—Description of access of paroxysm.—Appearance of the asthmatic in the height of the paroxysm.—Pulse.—Itching under the chin.—Muscular Phenomena.—Enlargement of capacity of chest.—Auscultatory signs.—Conclusions.

IN considering the phenomena of asthma, I shall take first the phenomena of a paroxysm, and then the phenomena of the disease generally; and I shall adopt this order because the phenomena of the paroxysm are so much more pronounced and marked, and constitute so much the body of the malady; while those of the disease generally, in opposition to those of the paroxysm, are rather the phenomena of the intervals, and consist of certain permanent conditions influencing the paroxysms, or produced by them.

As in epilepsy we have premonitory symptoms, in the form of the *aura epileptica*, spectra, and other subjective phenomena; then the establishment of the paroxysm; then those conditions of the nervous and muscular systems which constitute its climax; and then its abatement and the post-epileptic sleep;—so in asthma we have certain precursory symptoms, and then the attack in its accession, perfect establishment, and departure.

The precursory symptoms of a fit of asthma are liable to great variety in different individuals; some persons never experience any, but having been guilty of some imprudence, or the regular period of an attack having recurred, the seizure of the dyspnœa upon them is the first indication of its approach. But I think that the majority of asthmatics *do* know that an attack is coming on them, by certain feelings in themselves, or certain conditions of which they are aware. These symptoms generally show themselves on the night previous to the attack; but in some cases for a longer time. The patient will feel himself very drowsy and sleepy, will be unable to hold his head up, or keep his eyes open, and that without having undergone any particular fatigue, or done anything that could account for it.* I remember one case in which this was very strikingly marked; the asthmatic always knew when he was going to be ill the next day, by the

---

* Floyer was perfectly aware of this premonitory sign, having noticed it in his own person. "There appears," he says, "a great dullness and fullness of the head, with a slight headache, and great sleepiness on the evening before the fit."

extreme drowsiness that overpowered him at night; he would go sound asleep over his reading or writing, or whatever he might be engaged in, and that at an early hour of the evening. It was in vain for him to rouse himself; in spite of all his efforts, and in spite of the prophecies of those about him that he was going to be ill, and his own convictions of what awaited him, to bed he must go. And probably any resistance of these feelings would have been of no avail, and would neither have postponed nor modified the attack; the asthma was not the *result* of the heaviness, but the heaviness merely indicated the approach of the asthma; it was the commencement of that particular nervous condition of which the succeeding respiratory phenomena were but the more complete development; in fact, it must be looked upon as an integral part of the paroxysm. I find this precursory drowsiness to be the commonest of all the premonitory symptoms of asthma.

Others, again, know by extreme wakefulness, and unusual mental activity and buoyancy of spirits, that an attack awaits them; and I knew one case in which an attack of ophthalmia always ushered in the asthma: the man was liable to inflammation of his conjunctiva; it was always worse before his attacks than at any other time; and he invariably knew by the state of his eyes when he was going to suffer a paroxysm. It might be thought that this was a case of mere catarrh, that the asthma was caused by the inflammation of the eyes creeping down through the nasal mucous membrane into the air passages; but this was clearly not the case; there was no coryza, no bronchitis; the ophthalmia was strumous, and I believe that an exacerbation of the strumous cachexia—a more debilitated, and, therefore, a more irritable condition of system—was the cause alike of the inflammation of the conjunctiva and the spasm of the air-tubes. At other times, the precursory symptoms are connected with the stomach, and consist of loss of appetite, flatulence, costiveness, and certain peculiar uneasy sensations in the epigastrium; but here, I think, we have something more than mere premonitory signs. I think the relation of these symptoms to the spasm which follows, is often that of cause and effect.

Of all the circumstances attending the commencement of an asthmatic paroxysm, none is more constant than the time at which it occurs. This is almost invariably in the early morning, from three to six o'clock. There are some cases in which the usual time is the evening—some just after getting into bed, before going to sleep, and some in which there is no particular time; but the attack may come on at any hour of the day or night on the occurrence of some exciting cause, such as a fit of laughter, a full stomach, change of wind, &c. In nineteen cases out of twenty, however, the dyspnœa first declares itself on the patient's waking in the morning, or, what is much more common, wakes him from his sleep when he has had but half a night's rest.

Now, I think there are two reasons for the attack coming on at this time: one is, the horizontal position of the body; the other, the greater facility with which sources of irritation, and, indeed, any causes of reflex action, operate during sleep than during the hours of wakefulness. The first cause acts thus: when a person lies down and goes to sleep, the recumbent position favors the afflux of blood to the right side of the heart, and therefore to his lungs; in addition to this, the position of the body places the muscles of respiration at a disadvantage; add to this the diminished rate at which the vital changes go on during sleep; lastly, add to this the lowered sensibility of sleep, which prevents the arrears into which the respiration may be getting from being at once appreciated; and I think we have a sufficient explanation both of the time at which the attack generally comes on, and of the amount of dyspnœa that may accumulate before the asthmatic is roused from his slumbers. He goes to bed quite well, perhaps; the position of his body and the torpor of sleep soon throw his lungs into arrears, and they become congested; this goes on for some time, gradually increasing, without producing any particular effect; by and by, however, this pulmonary congestion reaches such a pitch that it becomes itself a source of great local irritation, and gives rise to asthmatic spasm; this, in its turn, cuts off the supply of air and increases the congestion, and thus the congestion and the asthma—the cause and the effect—mutually augment one another, till they produce such an amount of dyspnœa as is incompatible with sleep, and the patient suddenly wakes with all the distress of an asthmatic paroxysm full upon him. Now, in this case all the causes I have mentioned act together; but we know that each individually has its separate agency in producing the effect, because by removing any one of the causes, you may prevent the result; we know that the position of the body has to do with it, because an extra pillow may prevent the attack; we know that the disadvantage at which the muscles of respiration are placed during sleep has to do with it, because the attack may in some cases be prevented by laying the head on the arm, so as to make the shoulder a fixed point from which the accessory muscles of respiration can act.* Lastly, we know that the greater proneness to excito-motory action during sleep has to do with it; because some asthmatics do not dare to go to sleep after the commission of any imprudence, whereas they may be guilty of any irregularity with impunity if they only keep awake for some time afterwards. I know one asthmatic who often sits up half the night after taking a supper (breathing perfectly freely), because he knows that if he goes to sleep his asthma will come on him immediately; but by thus sitting up till his supper is fairly digested, his stomach empty, and the source of irritation thus removed, he may go to sleep fearlessly, and have a good night's rest.

One cannot help seeing the striking resemblance that exists between this and the orthopnœa of cardiac disease; only in the one case the extreme dyspnœa is brought about by the obstructed circulation through the lungs; in the other, by the sparing amount of air admitted through the obstructed bronchi; in both, the congestion of the lungs is first induced by the position of the body, and the sense of arrears—the *besoin de respirer*—blunted, and the respiratory efforts postponed, by the insensibility of sleep. But in the orthopnœa the violent and extraordinary respiration that succeeds the starting from sleep soon re-establishes the balance; whereas in the asthma the constriction of the bronchi, which persists after waking, precludes the admission of the necessary amount of air, and the dyspnœa remains.

---

* An asthmatic friend, with whose case I am familiar, tells me that he always sleeps much better on a sofa than a bed; no amount of bolstering can impart to a bed the comfort and ease of a sofa. This he attributes to the fixed support that the side of the sofa affords on which to rest his arm, and the leverage thereby furnished for the accessory muscles of respiration.

One curious circumstance with regard to time is, that it may be varied according to the intensity of the cause—the more intense the source of irritation the shorter will the sleep be before the asthma puts a stop to it. I once knew an asthmatic who was always awaked by his disease with an earliness proportionate to the size of the supper he had taken; certain airs disagreed with him as well as food before sleeping, and if the two causes acted conjointly he would wake with asthma much earlier than if they acted singly: thus if he went to a place that did not agree with him, he might wake about five o'clock with his asthma; the same if he eat a supper in a place that *did* agree with him; but if he eat a supper when staying at a place that did *not* agree with him, he would get no sleep after two or three o'clock; this may seem singular and an over-refinement, but it is strictly true; I have watched it over and over again.

How essentially characteristic of the disease this occurrence of the attack in the early morning is—how inherently a part of it—is shown by the fact that, in the great majority of cases, at this time, and at this time alone, will the attack come on, at whatever time in the twenty-four hours the exciting cause may be applied. For instance, in some cases over-exercise will bring on an attack: in many cases that have come under my care this has been so; but although the asthma was in these pretty sure to follow such over-exertion, it never came on immediately—never till the next morning; the exertion might be followed at the time by a little shortness of breath, not much exceeding that of a healthy person, which would speedily and entirely disappear, and the patient would pass the rest of the day, and go to bed, in perfect health; but, as surely as possible, he would be awoke the next morning at the usual time with his asthma. And it would make no difference at what time of the day the over-exertion had been taken, morning or evening; at the stated time and at that only, neither earlier or later, would its results declare themselves. Now, here we have an exciting cause actually and inevitably bringing on an attack, but powerless to do so, its effect suspended as it were, and laid dormant, until the characteristic time had come round. Nothing could show, as I think, more clearly than this, both the tenacity with which the disease sticks to its favorite time of occurrence, and its essentially nervous nature. For through what but through the nervous system could such exciting causes maintain their influence suspended, and, finally produce their effects after so long an interval, during which the respiratory and circulatory systems had been in a normal and tranquil condition?

I have always believed that this morning occurrence of asthma is the result of the causes I have mentioned,—the horizontal position and sleep, and the conditions of circulation and respiration that they induce; and I cannot but believe that this is its true explanation. But about six months ago a case came under my observation which seemed to imply that this feature of asthma was an essential part of its natural history, and not dependent on external circumstances. The case was that of a night porter, whose duties compelled him to turn day into night and night into day. He went to bed at seven o'clock in the morning, and slept through the early part of the day. But though the ordinary times of sleeping and waking were thus transposed, the asthma came on at the usual time, from five to six in the morning, towards the end of his vigil, when he was up and awake, and when none of the determining causes that I have mentioned could have been in operation. If the asthma had come on in this case at a time having the same relation to sleep and recumbency as in ordinary cases, it would have made its appearance about eleven or twelve o'clock in the day. This case certainly looks as if the particular period that the paroxysm affects depended on some inherent and inveterate habit of the disease. But the teaching of a single case like this is not to be taken in contravention of reason, or unsupported by further evidence. It is, however, I think, worth putting on record, and worth bearing in mind.

One of the symptoms frequently attendant on the first stage of an attack of asthma is *profuse diuresis* ; the patient will half fill a chamber-pot with pale, limpid water, exactly like the urine of hysteria. This abundant secretion generally comes on soon after the asthma commences; but I have known it come on so early that the patient was awakened from his sleep by the distension of his bladder, when the difficulty of his breathing was only just commencing. It generally lasts for the first three or four hours, and then ceases altogether. I believe the secretion of this abundant white urine to be of the same nature as the hysterical urine that it resembles—that it is nervous; and I regard it, as I have shown elsewhere,* as one of the many evidences of the nervous nature of asthma.

Another early symptom which I have often observed, is *neuralgic pains* —a deep-seated aching in the limbs and joints; the testicles, too, are very apt to be affected with it; and I knew one case in which the testicle and the tibia, from the knee to the ankle, were always affected on the same side, sometimes the right testicle and tibia only, sometimes the left, sometimes both; but always the tibia and testicle on the same side. The pain is constant, deep-seated, and wearying.

Let us now consider the phenomena by which an attack of asthma is generally ushered in. The patient goes to bed in his usual health, with or without premonitory symptoms; he goes to sleep and sleeps for two or three hours; he then becomes distressed in his breathing, and dreams, perhaps, that he is under some circumstances that make his respiration difficult; while yet asleep the characteristic wheezing commences, sometimes, without disturbing the patient himself, to such a degree as to wake those in the same or adjoining room, as if a whole orchestra of fiddles were tuning in his chest; perhaps he half wakes up and changes his position, by which he gets a little ease, and then falls asleep again, but only to have his distress and dreams renewed, and again partially to wake and turn. Shortly the increasing difficulty quite wakes him, but perhaps only for a moment or two; he sits up in bed in a miserable half-consciousness of his condition, gets a temporary abatement, sleep overpowers him, and he falls back, to be again awoke and again sit up; and so this miserable fight between asthma and sleep may go on for an hour or more, the dyspnœa arousing the sufferer as soon as sleep is fairly established, and sleep again overpowering him as soon as the wakefulness and change of position have a little abated the extremity of his sufferings. By and by the struggle ceases, sleep is no longer possible, the increasing dyspnœa does not allow the patient to forget himself for a moment, he becomes wide awake, sits up in bed to lie back no more,

* Med.-Chir. Review, July, 1858.

throws himself forward, plants his elbows on his knees, and with fixed head and elevated shoulders, labors for his breath like a dying man.

When once the paroxysm is established, the asthmatic offers a very striking and very distressing spectacle. If he moves at all it is with great difficulty, creeping by stages from one piece of furniture to another. But most commonly he sits fixed in a chair, immovable, unable to speak, or even, perhaps, to move his head in answer to questions that may be put to him. His back is rounded, and his gait stooping; indeed, his whole figure is deformed. His chest, back, shoulders, and head are fixed; he cannot even turn his head from side to side, but when he looks from object to object, merely turns his eyes, like a person with a stiff neck; his shoulders are raised to his ears, and his head thrown back and buried between them.

In order the better to raise his shoulders, and at the same time spare muscular effort in doing so, his elbows are fixed on the arms of his chair, or his hands planted on his knees, or he leans forward on a table, or sits across a chair and leans over the back of it, or he stands grasping the back of a chair and throwing his weight upon it,* or leaning against a chest of drawers or some piece of furniture sufficiently high to rest his elbows on in a standing position. At every breath his head is thrown back, his shoulders still more raised, and his mouth a little opened, with a gasping movement; his expression is anxious and distressed; the eyes are wide open, sometimes strained, turgid, and suffused; his face is pallid, and, if the dyspnœa is extreme and long, slightly cyanotic; the labor of breathing is such that beads of perspiration stand on his forehead, or even run in drops down his face, which his attendant has constantly to wipe. He is so engrossed with his sufferings and the labor of breathing, that he seems unconscious of what is going on around him, or else he is impatient, and intolerant of the assiduities of those who are in vain trying to give him some relief.

If the bronchial spasm is protracted and intense, the temperature falls; the oxygenation of the blood is so imperfectly performed, from the sparing supply of air, that it is inadequate to the maintenance of the normal temperature; the extremities especially get cold, and blue, and shrunk; I have known the whole body deathly cold, and resist all efforts to warm it, for four hours. But while the temperature is thus depressed, the perspiration produced by the violent respiratory efforts may be profuse, so that the sufferer is at the same time cold and sweating. It is this union of coldness and sweat, combined with the duskiness and pallor of the skin, that gives to the asthmatic so much the appearance of a dying man, and that even sometimes makes the initiated fear that death is impending.

The pulse during severe asthma is always small, and small in proportion to the intensity of the dyspnœa: it is so feeble sometimes that it can hardly be felt. The explanation of this is very simple. The imperfect supply of air produces capillary arrest—partial stasis—of the pulmonary circulation; but a small quantity of blood is therefore allowed to pass on to the left side of the heart, so that the volume on which the left ventricle contracts, and which it impels into the arterial system at each pulsation, is extremely

* I have known a patient stand in this position for two days and nights, unable to move.

small, and barely sufficient to register itself at the wrist. That the small pulse is due to pulmonary capillary arrest, itself due to the shutting off of air from the lungs, is proved by the fact that immediately the paroxysm yields, the pulse resumes its normal volume. I have never known the small pulse absent in severe asthma; its very explanation proves that it could not be.

One curious symptom of asthma, which I have found present in a large number of cases (I am not sure it is not universally present), but which I have never seen noticed in any treatise on the subject, is *itching under the chin.* I have often known that the breathing of asthmatics was tight, and told them so, from seeing them scratching and rubbing their chins. The itching is incessant, and of an indefinite, creeping character; but although it is impossible to help scratching it, the scratching does not relieve it. It is often accompanied with the same itching sensation over the sternum and between the shoulders, especially between the shoulders. It appears the moment the first tightness of breathing is felt, and goes off when the paroxysm has become confirmed—indeed I think it more pronounced in those slight and transitory tightenings of the breathing to which asthmatics are so liable (as, for example, after laughing), than in regular attacks. But I think it is the most strongly marked of all in the asthma that accompanies hay fever. The sternal and interscapular portion of this itching is, I think, of easy explanation, its distribution to the chin is less easy to understand. According to the law that the pain arising from the irritation of a viscus shall be referred to the superficies, front and back, in the middle line and at a level with the viscus (a law illustrated by the seat of the pain in stomach, bowel, and uterine disease), the seat to which the sensation from bronchial irritation is referred is the sternum and between the blade bones. Thus, in bronchitis, the raw, scraping feeling that accompanies cough is sternal and interscapular; so that in relation to this asthmatic itching, the fact would appear to be simply this—that while the impression on the bronchial nervous system produced by inflammation of its mucous membrane gives rise to sternal and interscapular *pain*, that produced by spasm of these tubes gives rise to sternal and interscapular *itching.* The itching of the chin must, I think, be of the same reflex character, and admit of the same explanation; but the reason of its locality is less apparent.

On stripping an asthmatic in the height of a paroxysm, an admirable example is seen of the immense array of muscles that become, on an emergency, accessory to respiration, and some idea is formed of the toil of the asthmatic, and the extremity of those sufferings that necessitate for their relief such intense labor. All the muscles passing from the head to the shoulders, clavicles, and ribs, are rigid, and the head is rendered a fixed point from which they can act on their respiratory attachments. Ordinarily these muscles, such as the splenii and scaleni, have their inferior attachments fixed, and move the head and neck; but now their upper attachment is fixed, and from it they act as mediate or immediate elevators of the ribs and distenders of the thoracic cavity; and this is how it is that the asthmatic is incapable of moving his head. By the contraction of the trapezius and levator anguli scapulæ, the shoulders are raised to the ears, in order that the muscles proceeding from the shoulders to the ribs may act at an advantage as elevators of these latter. The muscles of the back are so engaged in respiration, that they cease to support the trunk, and the gait becomes stooping. At every inspiration the sterno-mastoids start out like cords, and produce by their sudden prominence a deep pit between their sternal attachments. I have already referred to the gaping descent of the lower jaw at each inspiration.

Now, what is the explanation of this? What is its mechanism? I think the *rationale* of it is this: by its endeavors to raise the scapula, the omo-hyoid muscle is strongly contracted at each inspiration; but its hyoid attachment being by far its most movable extremity, the contraction of the muscle tends rather to draw the hyoid bone down than to elevate the shoulder; and as the elevators of the hyoid bone—the mylo-hyoid, genio-hyoid, and digastric—are firmly contracted with the view of fixing it, the drawing down of the hyoid bone also draws down the jaw, and thus is produced the descent of the jaw at each inspiration; so that this gasping movement really depends on one of the depressors of the hyoid bone being, by virtue of its scapular attachment, also an accessory muscle of respiration, and being at the same time, from the loose and floating character of its superior attachment, unable to effect that interchange of its fixed and moving points that takes place with regard to the other extraordinary muscles of respiration. In the case of other accessory muscles of respiration, either extremity can be made the fixed one, and thus render the action of the muscle respiratory or non-respiratory, according to circumstances; if the lower extremity is fixed, as is ordinarily the case, the head or neck is moved, and the muscle is non-respiratory; if the upper extremity is fixed, the shoulders or ribs are raised, and the muscle is respiratory. But the upper attachment of the omo-hyoid not being firmly fixable, the muscle cannot transfer its contractions to its respiratory extremity, and thus though theoretically, it is not actually, a respiratory muscle. This explanation, if correct, is not uninteresting, as it offers an example of the maintenance of a type of action in spite of disturbing circumstances that necessarily make the action inoperative; it is an instance, if I may so expr ss it, of morphological physiology, bearing the same relation to function as the retention, in obedience to type, of superfluous or modified appendages does to structure. I am not sure that the other depressors of the hyoid bone do not share in the action.

Meantime all the muscles that increase the capacity of the chest are straining their utmost and starting into prominence at each inspiration; as each breath is drawn, every muscle is thrown out into bold relief; and, since there are hardly any muscles of the trunk that are not mediately or immediately respiratory, the whole muscular system of the trunk may be mapped out in every part of its detail. The straining muscles are rendered all the more conspicuous from asthmatics being generally so thin.

But violent and laborious as are these respiratory efforts, they are abortive; although the muscles that should move the parietes of the chest are contracting to their utmost, no corresponding movements take place—the chest is almost motionless, its walls are fixed as in a vice, as if they could not follow the traction of their muscles; and this is really the case. This immobility, in spite of the violent action of the moving agent, is one of the most singular and striking appearances of asthmatic breathing. How different from the wide range of movement that follows even less considerable respiratory effort in one, to and from whose lungs the ingress and egress of air is free!

One result of these straining efforts to fill the chest is a permanent distension of it—its walls are kept fixed in a condition of extreme inspiration. So great is this enlargement of the chest during the paroxysm, that a waistcoat that would ordinarily fit cannot be brought together by two inches. But the chest is enlarged in every way, the diaphragm therefore descends, the abdomen therefore seems fuller, and its girth is increased. This, I believe, is the principal cause of that abdominal distension of which asthmatics complain, and which is generally assigned to flatulence. As soon as the paroxysm goes off, the chest and abdomen resume their original size. I do not see that anything is gained by this distension of the chest; the only difference is that the volume of air locked up in the chest is rather larger, but no more is changed at each respiration, and it is the amount so changed, and not the quantity contained in the lungs, that relieves the demand of respiration. Air is the thing that is wanted, and inspiration is the act that ordinarily relieves that want; this keeping the chest, therefore, at a condition of extreme inspiration must be looked upon as an instinctive, but blind and abortive, effort to remedy that which is irremediable.

Such being the external phenomena of the breathing of asthmatics, what are the auscultatory sounds that accompany it? They are exactly such as we should expect—exactly such as are consistent with these external phenomena, and such as imply, if the spasm is severe, an almost impassable bar to the ingress and egress of air. On applying the ear to the chest we hear—respiratory murmur *none;* and this is not because it is drowned by other sounds; if no other sounds are present it is equally inaudible; it is because the conditions of its production do not exist, because sufficient air is not admitted to generate it; just as there is no respiratory murmur in the long-drawn inspiration of whooping cough, or beneath thoracic parietes fixed by pleurisy or intercostal rheumatism. And this suppression of the ordinary breathing sound is a proof of the depressed standard at which respiration is being carried on, and of the completeness with which air is locked out of, and into the chest. The sounds that *are* heard are dry tube-sounds, large and small—rhonchus and sibilus of every variety, of every note and pitch, and in all parts of the chest, converting it into a very orchestra; but the sounds are mostly sibilant, high and shrill, resembling the chirping of a bird, the squeaking of a mouse, or the mewing of a kitten. And this smallness of sound makes me think that it is almost exclusively the smaller tubes that are the seat of the constriction, whilst the diffusion of the sounds all over the chest shows that constricted tubes exist everywhere.

There is one other fact, in relation to the sounds of asthma, that I think is instructive, and that seems to me to imply that the points of stricture are constantly changing their place, that spasm is constantly disappearing in one part and making its appearance in another; and that fact is, that the sounds are continually changing their character and site. On listening over a part of the chest where a few minutes before you heard a loud shrill sibilus, you find it gone; while a part that just before was silent is the seat of a chorus of piping. Now, if the sounds were of a moist character, if they were caused by mucus, I grant that such an inference could not be drawn, for sounds so caused may be, and constantly are, suddenly removed by cough, or other dislodgement of the accumulated secretion; but in the early part of an attack of uncomplicated spasmodic asthma there is no accumulation of mucus in the air-tubes—they are dry; the narrowing of the tube, therefore, that gives rise to the musical sound, being solely dependant on bronchial spasm, solely admits of removal by the relaxation of that spasm; and the frequent cessation and change of place of the pipings show that the spasm that causes them is transient and wandering.

The auscultation, then, of the asthmatic shows us these things:

α. The almost perfect stagnation of air in the chest, in spite of the violent respiratory efforts.

β. That the tubes are generally very small.

γ. That tubes in all parts of the chest are simultaneously affected.

δ. That the points of constriction are constantly changing place.

*British and Foreign Medico-Chirurgical Review.*

